# Is artists' perception more veridical?

**DOI:** 10.3389/fnins.2013.00006

**Published:** 2013-01-31

**Authors:** Florian Perdreau, Patrick Cavanagh

**Affiliations:** Laboratoire Psychologie de la Perception, Université Paris Descartes, Sorbonne Paris CitéParis, France

**Keywords:** artists, expertise, visual constancy, visual search, scene perception

## Abstract

Figurative artists spend years practicing their skills, analyzing objects, and scenes in order to reproduce them accurately. In their drawings, they must depict distant objects as smaller and shadowed surfaces as darker, just as they are at the level of the retinal image. However, this retinal representation is not what we consciously see. Instead, the visual system corrects for distance, changes in ambient illumination and view-point so that our conscious percept of the world remains stable. Does extensive experience modify an artist's visual system so that he or she can access this retinal, veridical image better than a non-artist? We have conducted three experiments testing artists' perceptual abilities and comparing them to those of non-artists. The subjects first attempted to match the size or the luminance of a test stimulus to a standard that could be presented either on a perspective grid (size) or within a cast shadow. They were explicitly instructed to ignore these surrounding contexts and to judge the stimulus as if it were seen in isolation. Finally, in a third task, the subjects searched for an L-shape that either contacted or did not contact an adjacent circle. When in contact, the L-shape appeared as an occluded square behind a circle. This high-level completion camouflaged the L-shape unless subjects could access the raw image. However, in all these tasks, artists were as much affected by visual context as novices. We concluded that artists have no special abilities to access early, non-corrected visual representations and that better accuracy in artists' drawings cannot be attributed to the effects of expertise on early visual processes.

## Introduction

Although most of us can hold a pencil and draw shapes on a surface, as we do in writing, only a few of us can use the pencil to accurately represent an object or a visual scene (Tchalenko, 2009). Why is there such a difference in drawing accuracy across subjects? According to Cohen and Bennett (1997), poor accuracy in drawing has several sources: misperception of the object, poor selection of the object's parts to be depicted, poor motor coordination, and misperception of the inaccuracies already present in a drawing. Their results suggested that misperceptions of the object might be an important contributor to poor drawing (Cohen and Bennett, 1997; Cohen and Jones, 2008). The artists' advantage may therefore lie in perceptual and cognitive skills that are more appropriate for the requirements of drawing: either a better, more vertical initial encoding of the object or subsequent, learned cognitive corrections. In this review, we will discuss both alternatives, and we describe our experiments (Perdreau and Cavanagh, 2011) that tested whether artists' perception is more veridical—closer to the retinal image—and less affected by the post-encoding corrections ordinarily made by the visual system: the so-called visual constancies. In the end, we argue that artists do not have any special perceptual expertise that would allow them to access to their retinal image or any learned strategies that recover that image. Instead, we suggest that the artist's advantage may arise from more robust representations of object structure in memory, representations that are more appropriate to the visuomotor demands of drawing.

### The artist's advantage

One of the most difficult tasks in **realistic drawing** is to convert a three-dimensional object or scene into a two-dimensional representation on a flat surface; that is, to represent the world as it is projected onto the retina, the back of the eye. Although visual processing starts with the retinal image, it ends with conscious perception only after a long chain of corrections and processes. The retinal image itself is constantly changing because of environmental variations in light or spatial position of the viewer. For example, the retinal projection of a far object is smaller than that of a near object, and the shape of an object on the retina changes with viewpoint and the amount of light it reflects changes with ambient illumination. However, we do not have this ever-changing experience of the world. Instead, we perceive a remarkable visual constancy, so that our final conscious percept roughly matches the external (***distal***) world's properties whatever the initial (***proximal***) retinal image (Todorovic, 2002).

These constancies help us function in the world, but an artist should not represent the world as looks to us, but rather as it appears on his or her retinas. For example, a distant car should be drawn smaller than a nearby child, even though we perceive the car as larger, and an object lying in a shadow must be drawn much darker than our subjective impression. How do artists undo the perceptual constancies in order to achieve their accuracy in drawing?

Numerous studies have investigated the possible origin of the difference in accuracy between artists and non-artists (e.g., Cohen and Bennett, 1997; Kozbelt, 2001; Cohen, 2005; Mitchell et al., 2005; Kozbelt and Seeley, 2007; Cohen and Jones, 2008; Matthews and Adams, 2008) and have reported a correlation between drawing accuracy and perceptual performances in many tasks. Notably, subjects who drew more accurately in these studies were less affected by visual contexts and visual constancies, making them “experts in visual cognition” (Kozbelt, 2001). Certainly the thousands of hours spent in training in visual arts create advanced skills, and this expertise may change the artist's visual processing. Strong effects of expertise on visual and memory processes have been reported in many other contexts (Hubel and Wiesel, 1970; Chase and Simon, 1973; Goldstone, 1998; Ostrovsky et al., 2006; Green and Bavelier, 2008).

However, it has not been established yet how this expertise enables visual artists to be less influenced by visual constancies, if indeed they are. Can they access an earlier, uncorrected representation of the object close to the retinal input—a modification of their visual processing compared to non-artists? Or do they use some learned strategies to later undo the corrections made by the visual brain? These two alternatives—direct access to the retinal image vs. later compensation for visual constancies—have long been disputed by art historians, especially by Ruskin (1912) and Gombrich (1960). According to the former, visual artists must train themselves with practical exercises and by using external tools (e.g., the Dürer's window) to access an “innocent eye,” not influenced by knowledge about the properties of the scene and the objects in it (e.g., shape, color, illumination, layout). This hypothesis has been strongly criticized by Gombrich (1960) who agreed that visual artists may use special techniques, but that those techniques do not provide better perception to artists. Instead, he argues that “making comes before matching” (Gombrich, 1960, p. 99), so that an artist would have to make a first draft and to modify it afterward by comparing it to the “original,” so that both are seen after visual constancies are applied (to the perception of the scene and separately to the perception of the drawing).

### Visual constancies

Visual constancies are corrections made by the visual system and allow a stable visual perception that attributes an object identity that is invariant to the environmental circumstances of viewing. However, the aim in realistic drawing is not to capture that invariance, but rather to make a snapshot of the physical world, where all the objects are observed from a given point of view at a certain distance from the artist, with specific illumination. In contrast, non-realistic drawing styles are not restricted to physical constraints and, as one example, are free to represent an object from different viewpoints simultaneously (e.g., cubism). In the following paragraphs, we will briefly describe examples of visual constancies and their underlying mechanisms that may be crucial for the experience of objects and surfaces that remain invariant to distance, viewpoint, and illumination.

#### Size constancy

**Figure 1:**
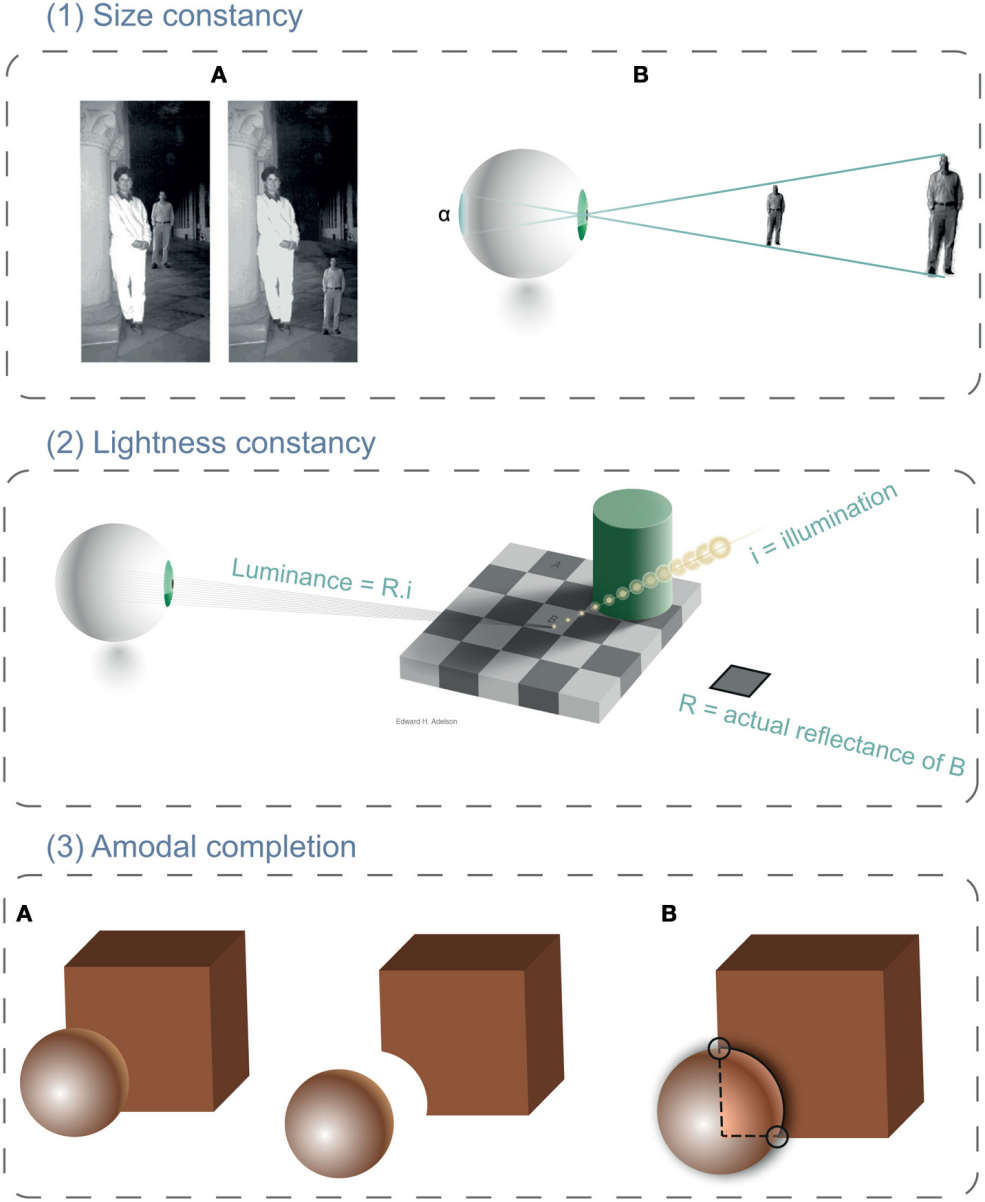
**Cases of visual constancies. (1) Size constancy.** In the left panel **(1A)**, we perceive the distant man as being as tall as the woman in the foreground. However, when the man is moved so that it appears to share the same distance with the woman (right panel, **1A**), he then looks much smaller than it was previously. This perception corresponds to visual constancy. Because the retinal sizes of the two images of the man (panel **1B**) are identical, the visual system assigns smaller size to the closer figure. **(2) Lightness constancy** (adapted from Adelson, 1993). Actually, squares A and B have identical luminance (same proximal property). However, because changes of luminance on the board's surface are attributed to a reduction of illumination due to the shadow casted by the cylinder, also a similar change in luminance is attributed to B's surface. Thus, the visual system automatically compensates for this change and makes B appearing lighter than A. **(3) Amodal completion**. Panels in **(3A)** can be interpreted either as a sphere occluding partially a cube or as a notched cube touching a sphere. This latter is harder to see, for the presence of T-junctions **(3B)** is taken as cues for occlusion and depth by the visual system. Hence, the brain extends the contours (dashed lines) to join them and to form a complete cube.

The car's size on our retina will decrease as the car moves away from us. Yet, instead of perceiving a car getting smaller we see it as having the same size. This is size constancy. This ability to accurately judge the size of an object despite changes in retinal size (in visual angle) is attributed to corrections made by the visual system for the perceived distance. But precisely because it depends on distance perception, it is also affected by cues to depth used by the brain to estimate distance (Leibowitz and Harvey, 1969; Day, 1972; Todorovic, 2002). Such correction, for example, can lead to size illusions when two objects having the same size in a 2D image appear to be at two different distances. In this case, the apparently closer object appears smaller than the farther one (see Figure 1 Stuart et al., 1993; Aks and Enns, 1996; Bennett and Warren, 2002). Nevertheless, when asked, subjects can attempt to judge the distal size of an object (how big is that car over there) or to judge its retinal size (would it be as big as my thumb if I held it out to block the car). However, Rock (1983) showed that even when instructed to judge retinal size and shown how to do it, the judgment is still biased toward the distal, “perceived” size (Carlson, 1960, 1962).

Because an artist has to depict a 3-D scene on a 2-D canvas, he or she has to employ pictorial cues to capture the illumination and depth in the scene, cues such as reflections, shadows, relative size, linear perspective, and elevation in the field (see in particular Solso, 1996). Moreover, embedded in these cues are the depictions of objects that should match their retinal size and luminance, if properly captured, as if the artist were able to see them directly, independently of the perceptual corrections that then act on them. Our recent paper (Perdreau and Cavanagh, 2011) asked whether, after years of practice drawing distant objects as smaller than nearby ones, as they are on the retina, do visual artists judge the retinal size of objects any better than non-artists.

#### Brightness constancy

Size constancy is only one of the corrections made when we perceive the objects around us. We also correct for the illumination falling on them. Objects are perceived through the light they reflect back to our retina. However, the light falling on the retina (the *proximal* stimulus) is the product of the object's surface **reflectance** (the *distal* stimulus) and the ambient illumination (also *distal*) falling on the object's surface (Figure 1). Lightness corresponds to the perceived reflectance (white vs. black or light vs. dark) and brightness designates the perceived **luminance** (Adelson, 1993). For example, the snow is rarely white but more often blue or brown because of the light coming from the sky or from trees. But, we ordinarily see it as white. Our subjective percept of the snow's color (the perceived reflectance) remains invariant whatever the ambient illumination (Gilchrist, 1988; Moore and Brown, 2001). Several studies described mechanisms explaining how the visual system would estimate and discount the illumination to recover the actual surface reflectance (Gilchrist, 1988, 2006; Arend and Spehar, 1993a,b; Adelson, 2000; Agostini and Galmonte, 2002). Although lightness constancy has attributed to **low-level** mechanisms (e.g., a simultaneous contrast effect due to lateral inhibition of retinal ganglion's cells), in fact, high-level processing of spatial configurations of surfaces and light sources is required. For example, if surfaces are seen within a cast shadow, which decreases the luminance within its limits, the visual system will attribute the change of luminance due to the shadow to a change in illumination and not a change in reflectance (Gilchrist, 1988; Cavanagh and Leclerc, 1989).

In paintings, the artist can only vary the reflectance of the canvas' surface, and thus he or she must pick the appropriate pigment to match the actual object's luminance. Thus, according to Ruskin, an artist “sees the colors of nature exactly as they are, and therefore perceives at once in the sunlighted grass the precise relation between the two colors that form its shade and light” (Ruskin, 1912, p. 4). Our recent paper also asked whether artists can better judge the actual object luminance by ignoring ambient illumination.

#### Amodal completion

Amodal completion, another case of perceptual constancy (Rock, 1983), corresponds to an inference or percept of a complete object even though the retinal input of that object is only partial. For example, when a square is partially occluded by a circle, we still perceive it as a square behind a circle and not as a notched square (see Figure 1 Kanizsa, 1979, 1985). This completion effect has long been described in terms of Gestalt configuration laws (e.g., collinearity, Kellman and Shipley, 1991) which are linked to low-level mechanisms (e.g., edge detection, orientation in V1, and solving of “border-ownership” in V2 complex cells Bruno et al., 1997; Rensink and Enns, 1998; Tse, 1999; Wolfe and Horowitz, 2004). Shape analysis has been described as a sequence of stages from edges to parts (*mosaic* stage) to the whole (*completion* stage) that produces a viewpoint invariant percept (e.g., Biederman, 1987). But surprisingly, it has often been demonstrated that the whole, the highest level representation, is accessed earlier than the parts (He and Nakayama, 1992; Rensink and Enns, 1998; Hochstein and Ahissar, 2002; Wolfe and Horowitz, 2004). These results imply that object-level representations are the first to be represented in conscious perception, and that it takes longer to access the object's parts because the visual system has to “unbundle” them (Tse, 1999; Hochstein and Ahissar, 2002; Lee and Vecera, 2005). The third question we asked was whether visual artists can ignore the completed form of an object's representation and access the “mosaic” image of its parts (as they are on the retina) and do this more quickly than non-artists?

### Perdreau and cavanagh, 2011

Our earlier paper addressed whether visual artists develop expertise in visual cognition that includes access to earlier visual representations unaffected by visual constancies. We ran three experiments testing size, lightness constancy, and amodal completion. The first two experiments (size and lightness constancies) use a matching-to-standard paradigm while the last experiment (amodal completion) was a visual search task. In all of these tasks, visual constancies were introduced with the use of visual context, perspective grids, cast shadows, and occlusion (Day, 1972; Todorovic, 2002, 2010).

## Methods

**Figure 2:**
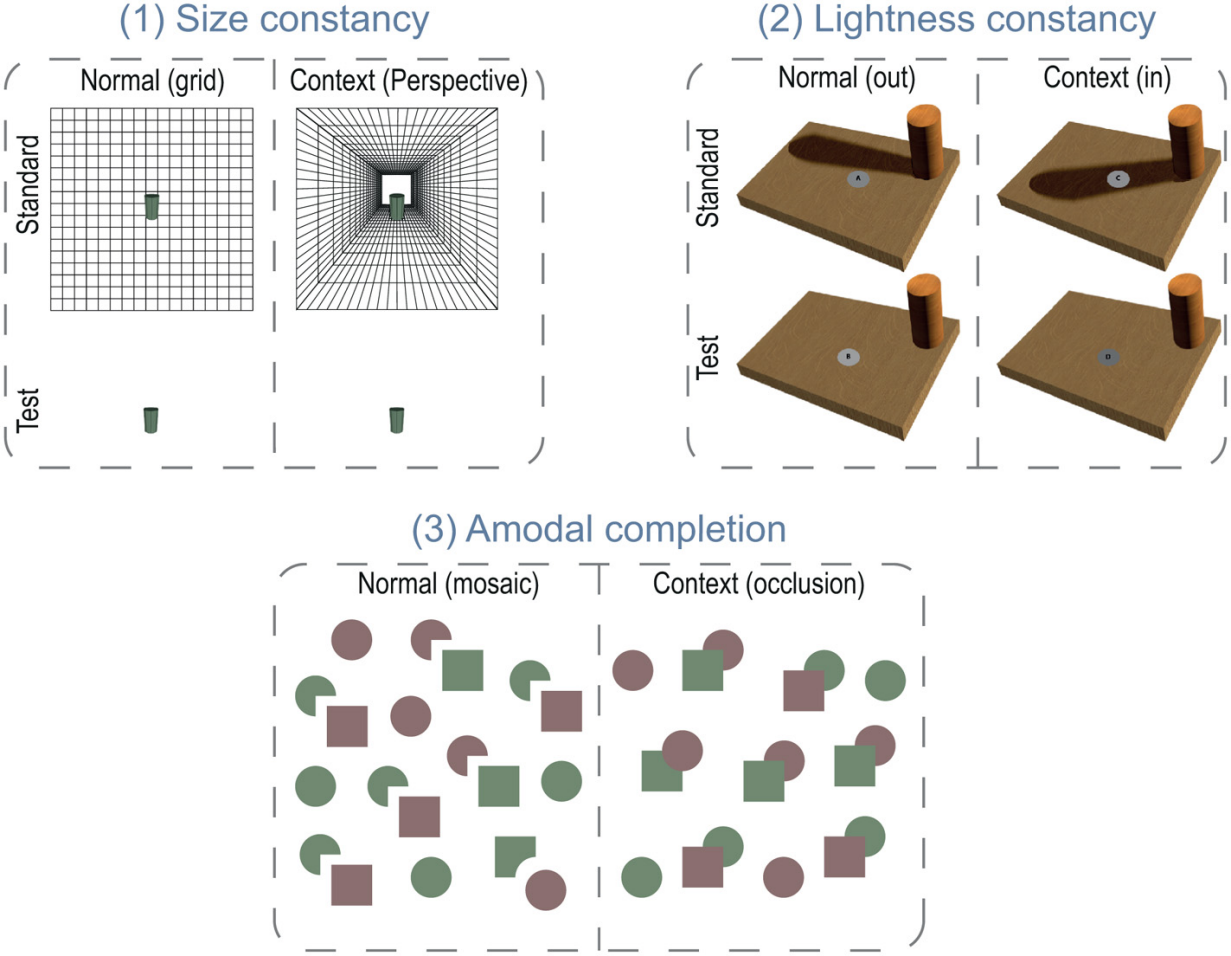
**Visual constancies tasks. (1) Size constancy task.** Subjects were asked to adjust the height of a “test” cylinder so that it matches the “standard” cylinder's size, which could be either on a simple grid (normal) or a perspective grid (context) background, and were instructed to make their settings as if they were measuring the cylinder with their fingers on the screen. **(2) Lightness constancy task.** Subjects were instructed to match the “test” patch's luminance to the actual “standard” patch's luminance, and were explicitly told to ignore the presence of the cast shadow (context) surrounding the standard patch, as if they were looking through a tube and seeing only the gray patch. The standard patch could be either outside a cast shadow (normal) or within it (context). **(3) Amodal completion task.** The task was to search for a notched square among squares and circles and report the notched square's color (red or green). Some of the circles also had notches. In the normal (mosaic) condition, the target was far from its companion circle, as were the Pacman-like, notched distractor circles, and their companion squares. In the context condition, the notched square abutted its companion circle, and the notched circles abutted their companion squares, so that the shapes appeared completed behind the overlapping shapes. In particular, the target appeared to be a full square partially hidden behind a circle, camouflaging its notched shape.

Methods commonly used in research studies of size, shape, and brightness constancies can be categorized in two kinds: drawing tasks (e.g., Thouless, 1931; Lee, 1989; Mitchell et al., 2005) and matching-to-standard tasks (e.g., Carlson, 1966; Leibowitz and Harvey, 1967). In drawing tasks, subjects have to draw a target stimulus presented within a specific visual context (e.g., perspective). This technique is not appropriate to study the impact of visual constancies on an artist's perception, since it will also involve visually guided motor skills that are central to the artist's domain of expertise. For that reason, we used the adjustment technique and presented both the *standard* and the *test* stimuli simultaneously and without a time limit (Figure 2).

Our tasks required our subjects to adjust the size (size task) or the luminance (lightness task) of the standard stimulus to match the retinal image and not the perceived property of the standard stimulus. This is problematic because subjects do not have an intuitive sense of “retinal size” or “received luminance” as these are usually corrected by visual constancies into perceptions of object size or object surface color (Carlson, 1966; Leibowitz and Harvey, 1969). To make these judgments of “proximal” values more direct and intuitive, we recast the tasks to focus on the proximal values. Instructions were “Please, make your adjustment so that the height of the test cylinder corresponds to that of the standard cylinder. Do it as if you were measuring the cylinder's size with your fingers and only focus on the physical cylinder displayed on the screen.” For the lightness task, we asked the subjects to “make the test patch's surface lighter or darker to match it with the standard patch's surface. In particular, imagine that you could look at the surfaces through a tube and ignore the presence or absence of the surrounding shadowed region.”

Both the size and the lightness tasks used a matching-to-standard task. The screen was divided in two halves, the upper being the *standard* part, the lower the *test* part (Figure 2). For the size task, a “*standard*” cylinder (upper part) stayed over a background that could be either a black line-drawn grid simulating a wall (normal condition) or a black line-drawn central perspective grid corresponding to a hallway (context condition). Such a perspective display includes several monocular cues to depth: linear perspective, elevation-in-the-field, and texture gradients (Ames, 1925; Bennett and Warren, 2002) that affect distance perception. Thus, a cylinder lying on it may appear more distant than the test stimulus that only lies on flat wall grid. Therefore, for the same retinal size, the cylinder would appear larger, unless the subjects were able to ignore this context. In the brightness task, the two parts presented identical boards textured with a wood surface with a wood cylinder standing on it. On the *standard* board (upper), the cylinder cast a shadow that could either cover the standard patch (context) or falling beside (normal). On the test board, there was no cast shadow.

Finally, to disentangle whether the artist's advantage comes from an uncorrected initial perception (Ruskin, 1912), as compared to the use of strategies that undo constancies after the fact (Gombrich, 1960), we used a visual search task adapted from Rensink and Enns' experiment (Rensink and Enns, 1998). This task allowed us to measure the processing speed of each item in the search array. This processing speed can differentiate an automatic, parallel, and fast analysis from an attentive, serial, and slow analysis (Treisman and Gelade, 1980). In our task, the target was a green- or red-notched square that was accompanied either by a distant circle (normal) or by a contacting circle (context). The distractors (a different number on each trial, ranging from 2 to 12) were green or red circles with a missing quarter accompanied by distant (normal) or contacting (context) squares (Figure 2). The subjects' task was to report the target's color as quickly and accurately as possible. When the target, the notched square, was in contact with its adjacent circle, it would appear to be a complete square partially occluded by a circle (He and Nakayama, 1992; Rensink and Enns, 1998), making it quite difficult to find among all the actually complete, distractor squares. If artists can access earlier representation of shape that would be unaffected by amodal completion the notched square should be very distinctive among all the complete squares, making it easy to find no matter how many distractors are present (parallel processing). However, if they do not have access to the raw image, each square-like shape has to be scrutinized individually to discover the notched one and the processing rate (the increase in reaction time with the number of distractors) will be quite slow.

For these three tasks, we divided our subjects into three distinct groups: non-artists, art students, and professional artists. The 14 non-artists were recruited from a database of voluntary subjects and they reported that they had no training in any visual arts [9 females, mean age: 23]. The nine art students were recruited from the École des Beaux Arts in Paris [6 females, mean age: 22 years]. Finally, the 14 professional artists were recruited from galleries, workshops, and international artists associations [9 females, mean age: 39 years]. At the beginning of the experiment, we first gathered information about each subject's training in visual arts. Then, each ran the three tasks in a random order. All the art students and professional artists reported having a formal training as well as continuous practice in visual arts. In particular, their training included observational drawing and painting. Over the three groups, experience in visual arts ranged from 0 (novices) to 41 years.

## What happened

This section summarizes the results we found in our earlier study and details about the statistics may be found in the corresponding paper (Perdreau and Cavanagh, 2011).

**Figure 3:**
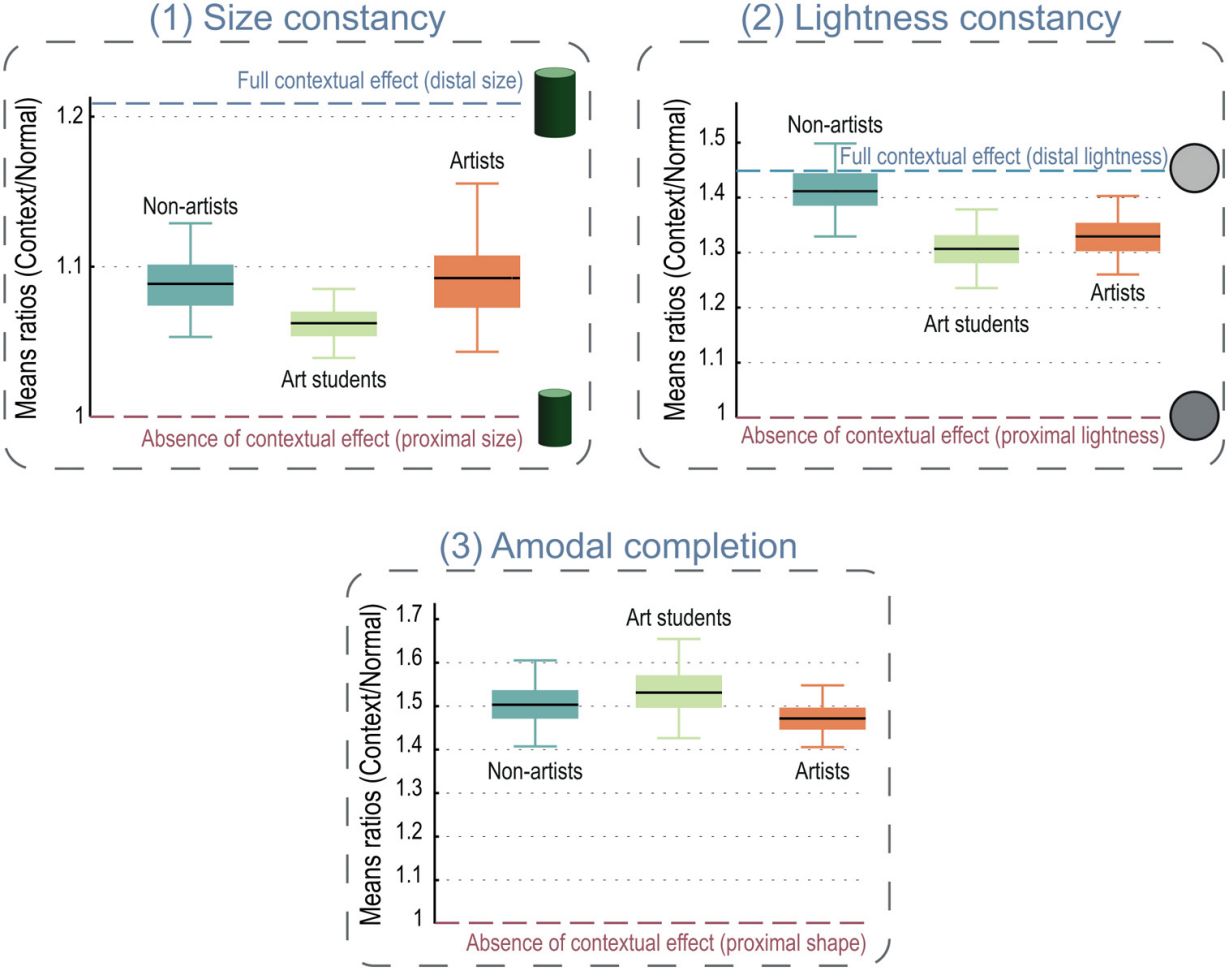
**Mean ratios in the three experiments.** The expected performance if subject could access the proximal value (the retinal image) uncorrected by visual constancies is given by the ratio of “1” on each graph. Error bars are 95% confidence intervals. The graphs show the proximal value for the stimulus on the right at the ratio of 1 and estimates of the proximal values for size **(1)** and lightness **(2)** only. **(1) Size constancy**. There were no difference between groups and they all showed an effect of the perspective on their perceptual judgment (ratios > 1). We estimated the distal size by assuming a slant of 20° and so a distance to the cylinder of 58 cm vs. the screen's distance of 52 cm (estimates based on derivations in Todorovic, 2002). Distal size was therefore about 21% greater than the proximal size. **(2) Lightness constancy**. Similarly, all the groups were strongly affected by the presence of the cast shadow, and artists showed no advantage in this task. Distal luminance (lightness) was estimated by adding the decrease in luminance due to the cast shadow (about 45%) onto the proximal luminance. **(3) Amodal completion**. Artists were not better or faster at accessing the proximal shape of the target (notched square). All the groups were strongly influenced (about 50% of context effect) by amodal completion triggered by the contact between the target shape and its companion circle. Even where visual artists showed a numerical (but non-significant) advantage over non-artists **(2)**, it was far from enough to completely undo the visual constancy (ratio = 1) and so cannot fully explain the artist's accuracy in drawings.

In both the size and the lightness tasks, we computed the mean settings for both the context and the normal conditions and looked at the ratios of the mean CONTEXT response over the mean NORMAL response (Figure 3). All the ratios were significantly greater than 1, indicating that the subjects' judgments were strongly affected by the visual context. Nevertheless, we found no significant differences between the groups' ratios. In both tasks, the artists' judgments were just as affected by the contexts as those of the non-artists. There was no effect of expertise on perception. Moreover, the correlation between the subjects' years of experience in drawing and the context's effect on their perceptual judgments was not significant.

Finally, to evaluate the effort put by the subject to perform the task, we analyzed their response times in the size and lightness tasks. Interestingly, we found that art students and professional artists spent significantly more time to make their judgments, taking, respectively, 15.4 and 15.9 s against 8.6 s for the non-artists in the size task, and, respectively, 11.4 and 14.3 s against 9.7 s for the non-artists in the lightness task.

In the visual search task (amodal completion), subjects' slopes were significantly higher in the *context* condition than in the *normal* condition, indicating a strong effect of the context on the subject's search speed. Similarly to the two other tasks, we computed ratios between the mean response time in the context and the normal conditions (Figure 3) and all of them were significantly greater than 1 showing a robust slowing of the search due to the amodal completion. We again found no significant difference between the groups indicating that artists had no better access to the raw image where the notched square would have been easy to find. Finally, the correlation between subjects' experience and the effect of the context was not significant.

## Discussion

Our everyday perception needs to allow us to act on our environment. This requires a stable visual perception of objects that are the goals of our actions. This perceptual constancy is given by the appropriate corrections made by the visual system for changes in distance, lighting, and viewpoint (Hochstein and Ahissar, 2002; Ahissar and Hochstein, 2004), the so-called visual constancies. However, acting on the world is not the artist's aim when he or she wants to represent it. Instead of perceiving a high-level description of the world, they need to access low-level details (lines, orientations, visual directions). One could reasonably expect that years of training in realistic visual arts might modify the functional organization of the visual system and allow artists to access these low-level, early descriptions more directly. Indeed, some studies reported that more skilled subjects in drawing outperformed novices in perceptual tasks (e.g., mental imagery, object recognition, visual search for embedded figures, and Gestalt completion (Kozbelt, 2001; Calabrese and Marucci, 2006), and that they showed a lesser influence of shape constancy in drawing and perceptual tasks (Thouless, 1932; Cohen and Bennett, 1997; Mitchell et al., 2005; Cohen and Jones, 2008). One reason for the greater accuracy of artists could be that they can access the raw image of an object, less influenced by high-level corrections. However, it remains unclear whether visual artists can really access a proximal representation, and if this access is direct or supposes some strategies to undo the visual constancies afterward. Our results (Perdreau and Cavanagh, 2011) suggest that artists have no special abilities to undo visual constancies and must deal with these automatic corrections as any normal observer would.

Specifically, all the subjects in the three experiments showed a strong effect of the context on their perceptual adjustments, ranging from 7 (size) to 50% (amodal completion) with no advantage for the artists. In one task (lightness), visual artists (students and professional) were numerically less influenced by the presence of a cast shadow in the luminance judgment, but they still showed an effect of context of at least 30%, which is far from the 0% needed for photorealistic drawing. Interestingly, in both the size and the lightness tasks, art students and professional artists spent much more time to make their setting. If visual artists were able to access an earlier, uncorrected representation of the object, this task would have been easier and faster for them than for non-artists. Our results suggested that artists could not access the standard stimulus' property (size or luminance) directly. Moreover, because our method allowed the subjects to make continuous adjustments of the test stimulus size or luminance, it allowed them to make all the corrections they felt necessary—as they would do in a drawing task. This produced no increased accuracy in their performances, strongly suggesting that the artists' advantage cannot be explained by perceptual factors alone. The visual search task's results were in line with this finding, for artists showed as the same effects of context—the same slow-down for the camouflaged targets—as the non-artists. This echoes Kozbelt's study (2001) that showed that, when predicting drawing accuracy from perceptual performances, residuals of the regression still distinguished artists from novices, suggesting that non-perceptual processes such as visuomotor skills might explain the artist's advantage.

Although we found no perceptual advantages for artists in our experiments, earlier studies have reported some advantages. For example, a number of studies showed that skilled draftspersons outperformed novices in shape constancy tasks, a task that we did not examine (Thouless, 1932; Mitchell et al., 2005; Cohen and Jones, 2008). However, recent studies failed to replicate those findings (McManus et al., 2011; Ostrofsky et al., 2012). Nevertheless, Ostrofsky et al. (2012) did report an advantage for artists in a size constancy task where we had found none. Their artists showed a 15% effect of context vs. a 20% effect for the non-artists. Their size constancy task used more monocular cues to depth than ours (e.g., shading) and both the standard and the test stimuli were presented within the same scene in contrast to our experimental settings. These differences may have increased the effect of context on distance perception, and this may explain some of the effect they find. Whatever its source, this decrease from 20 to 15% influence of context on size judgments in the Ostrofsky et al. study is far from the absence of effect (0%) required for veridical representation, as suggested by the authors and by Ruskin's innocent eye hypothesis (Ruskin, 1912). However, as stated in previous studies that found a link between drawing accuracy and perceptual errors made in a visual constancy task (Mitchell et al., 2005; Cohen and Jones, 2008; Ostrofsky et al., 2012), a more likely hypothesis would be that a reduction of constancy, and not a total absence, is enough to result in a more accurate drawing.

Nevertheless, what could be the source of these discrepancies across studies? Indeed, even studies using the same method did not find similar results (e.g., Cohen and Jones, 2008; McManus et al., 2011). One possible factor is that the evaluation of drawing accuracy is often done by non-experts according to subjective criteria. A better approach would be to use more objective measurements such as geometrical and physical properties of the drawing itself (e.g., Mitchell et al., 2005; Carson et al., 2012).

If our results do not prove the absence of difference between artists' and novices' perceptual performances in our tasks, so that it remains plausible that artists could be less influenced by visual constancies, the results of the visual search task, however, argue against the hypothesis of a direct, veridical perception in visual artists (Ruskin, 1912). Indeed, artists' processing time, and hence the time needed to access the target representation, were as long as that of novices. Although the better accuracy of artists in drawing is part of what makes them artists, one cannot attribute it solely to perceptual expertise. Moreover, the fact that our artist subjects took almost twice the time to perform our matching tasks (size and luminance) than our non-artists suggests that they attempted to apply some strategies to overcome the effect of visual constancies, strategies that are often part of an artist's training. One could argue that they were more motivated by this challenge to their specialized abilities, but so, this was in vain, since they were as much affected by context as non-artists. One explanation for the veridicality of artists' representations, of the seeming independence from visual constancies, is Gombrich's model of schemata (Gombrich, 1960). Visual artists are like copyists: they must start with the same corrected and biased perception as anyone, but they apply it to both the scene and their drawing. They then correct progressively their sketch so that it matches the scene they are painting. This latter hypothesis is closer to descriptions of how artists work (Locher, 2010). This mode of drawing—matching the percepts in the original and on the drawing—does not make artists any better at discounting the visual constancies. Therefore, since neither approach (perceptual vs. cognitive) improved the artists' access to the veridical retinal image, we must assume that special expertise in perception of the scene, either directly or following cognitive corrections, is not the source of artists' accuracy.

Finally, as suggested in our earlier study (Perdreau and Cavanagh, 2011), this absence of difference between artists and non-artists could be due to the nature of our tasks. They were indeed only perceptual and might have not required mechanisms ordinarily called on during the drawing process. A recent study suggested, however, that artist's advantage is not domain-specific but might transcend the requirements of drawing, so that artists become expert in visual cognition in general and, as such, they might outperform novices in perceptual tasks related to drawing (Glazek, 2012).

Our results suggest that the artist's advantage is not based on a direct access to early visual representations. As an alternative, we have begun to examine whether their accuracy arises from the way they analyze and represent an object's structure. In particular, the drawing process is never completed all at once but requires iterative processing of the stimulus as the drawing progresses. This is reflected by many eye movements alternating between the “original” and the “copy,” where the eye movement targets are highly dependent on motor constraints of the drawing hand—its current position on the drawing and where it can move next (Tchalenko and Miall, 2008; Coen Cagli et al., 2009). This may indicate that to render an object accurately, one dissects it into smaller parts for reproduction while respecting the spatial organization within and between all the object's parts in order to capture the object's spatial layout and proportion. Thus, our current hypothesis is that years of practice in visual arts lead artists to better encode each object's structure in order to facilitate its reproduction. This is guided by a better understanding of what should be selected and encoded from the object, such as structural features (junctions or vertices, Kozbelt, 2001; Biederman and Kim, 2008; Kozbelt et al., 2010; Ostrofsky et al., 2012) and their spatial positions and relationships. Above all, this expertise needs to be optimized in a form that best guides the final motor production. The structure of the original encoding should ultimately respect the visuomotor mapping required to produce the final tracing (Seeley and Kozbelt, 2008; Tchalenko, 2009; Glazek, 2012).

### Conflict of interest statement

The authors declare that the research was conducted in the absence of any commercial or financial relationships that could be construed as a potential conflict of interest.

## Key Concept

Realistic Drawing: Here, we use the term “realistic drawing” to refer to any visual arts, whatever the medium, having the aim of representing the world as accurately as possible. It thus concerns practices such as illustration, painting, or drawing (in color or not).
*Distal*: The term “distal” corresponds to actual properties (e.g., color, size, or shape) belonging to the external world, independent from the observers.
*Proximal*: “Proximal” refers to the retinal domain. As such, it is still external, that is non-relative to a subjective percept.
Reflectance: The reflectance is the proportion of incident light reflected at different wavelengths of the spectrum and fully depends on the surface material. It is an object's intrinsic property that is independent from the intensity and the wavelength of the ambient illumination, and that gives, for example, the surface color.
Luminance: The luminance is the product of the illumination falling on the object's surface and the surface's reflectance.
Low-level: Low- vs. high-level processing refers to “bottom-up” vs. “top-down” processing. Low-level mechanisms depend directly on the sensory inputs and do not need higher cognitive processes (e.g., extracting shape from the integration of lines and contours). In contrast, high-level mechanisms call on a variety of cognitive operations (e.g., hypotheses made by the system based on object and scene properties).
